# Lack of Hydraulic Acclimation in Response to Multiple Droughts and Recovery

**DOI:** 10.1111/pce.70671

**Published:** 2026-06-15

**Authors:** Jaycie C. Fickle, Kelly L. Kerr, Lillie I. Congram, Cedric Zahnd, William R. L. Anderegg

**Affiliations:** ^1^ School of Biological Sciences The University of Utah Salt Lake City Utah USA; ^2^ Department of Ecology, Evolution, and Marine Biology University of California Santa Barbara Santa Barbara California USA; ^3^ Wilkes Centre for Climate Science and Policy University of Utah Salt Lake City Utah USA

## Abstract

Human‐caused climate change is expected to bring more frequent and extended droughts with shorter wet periods of relief in between for many regions of the world. Critical knowledge gaps remain around the extent to which tree species can acclimate physiologically to repeated droughts. Using a common garden experiment with saplings of the widespread and ecologically important trembling aspen (*Populus tremuloides*), we studied how repeat drought and recovery treatments impact plant functional traits and physiological responses. We utilised four treatments over the course of 3 years: a continuous control, a three consecutive drought year treatment, a two consecutive drought year treatment, and a treatment with two drought years with a well‐watered recovery year in between. Trees that experienced a recovery period between two drought treatments exhibited less drought resilience compared to both the two and three‐consecutive‐year treatments. Trees in the recovery treatment also exhibited lower growth rates and increased xylem damage in their older rings. Additionally, we observed no evidence of hydraulic acclimation to severe drought over time. These findings indicate that, during severe and repeated droughts, aspen saplings are likely to experience increases in hydraulic vulnerability, and wet periods between droughts are not likely to improve overall drought responses.

## Summary statement

Aspen saplings (Populus tremuloides) which experienced a recovery period between drought treatments exhibited less resilience compared to both the 2 and 3‐consecutive‐year treatments. We also found no evidence of hydraulic acclimation to drought over time, but some evidence of damage accumulation.

## 1 Introduction

Over the 21st century, anthropogenic climate change is projected to result in more frequent drought events and higher temperatures for many regions worldwide, likely further driving large‐scale forest die‐offs (Allen et al. 2010; IPCC 2014; Spinoni et al. 2018; Grossiord et al. 2020). More frequent droughts may result in repeat, multi‐year droughts that extend through multiple years, with less frequent wet years in between (Dai 2013; Cook et al. 2015). Given that even single‐year drought events can cause legacies of damage and mortality lasting for several years (Anderegg et al. 2013), multi‐year droughts may pose an even bigger challenge to trees' ability to withstand water stress (Moravec et al. 2021; Rakovec et al. 2022). Western North American trembling aspen (*Populus tremuloides*) forests have been particularly impacted by repeat droughts in the past decades, with many aspen stands across the western US undergoing drought‐driven sudden aspen decline (SAD; Worrall et al. 2010). SAD includes a range of symptoms from the loss of leaves and branches to the total mortality of the overstory, with typically little regrowth once forests have died (Worrall et al. 2008; Worrall et al. 2015; Worrall et al. 2008; Worrall et al. 2010; Love et al. 2019). It has been observed for many angiosperm tree species, including aspen, that once trees have experienced hydraulic damage from a drought, trees are weakened and may be more susceptible to future droughts (DeSoto et al. 2020). However, trees may also acclimate physiologically or morphologically when experiencing a drought, potentially making them more resistant to future droughts (Gessler et al. 2020). It is therefore important to understand how physiological damage can accumulate to lethal levels over repeat droughts, and the potential for trees to acclimate to and recover from droughts (Anderegg et al. 2014; Anderegg et al. 2020; Fajardo and Piper 2021; Limousin et al. 2022).

Drought‐induced damage to the xylem is one of the primary mechanisms mediating SAD (Anderegg et al. 2013). During drought, xylem is at risk of developing emboli which block off water transport and spread from one vessel to another in a process termed cavitation (Sperry and Tyree 1988). Irreversible damages occur during this spread of emboli, where the emboli stretch and tear the inter‐vessel pit membranes (Hacke et al. 2001; Hillabrand et al. 2016). These damages cause cavitation fatigue, which reduces xylem resistance to emboli because the pit membrane pores become “leaky”, allowing easier spread of embolism from one vessel to another (Hacke et al. 2001; Anderegg et al. 2013). Cavitation fatigue limits recovery, growth, and can lead to mortality (Anderegg et al. 2013). Additionally, once undergone, trees are more vulnerable to subsequent droughts, even low‐severity drought events (Anderegg et al. 2013; Hillabrand et al. 2016; Anderegg et al. 2020). There is some evidence that embolized vessels can refill, at least in some species and under certain conditions, such as under positive pressures and after freeze‐thaw cycles (Klein et al. 2018; Cochard et al. 1994; Sperry et al. 1994; Davis et al. 1999; Love and Sperry 2018). However, cycles of refilling in vessels that have already been weakened by drought stress result in increased damages (Hacke et al. 2001) and hydraulic capacity is sometimes only restored with new xylem growth (Brodribb et al. 2010; Gauthey et al. 2022).

Recovery from drought and the potential for acclimation are currently large knowledge gaps in our understanding of tree drought response. Many factors can impact how well trees recover from drought events, including the timing, duration and severity of the drought, and morphological and physiological aspects of the individual or species (Miao et al. 2009; Gessler et al. 2020; Huang et al. 2018; Fajardo and Piper 2021; Limousin et al. 2022). Additionally, soils are sometimes not able to refill moisture content to pre‐drought levels due to changes in various soil physical and chemical properties (Muhr et al. 2010; Huang et al. 2018). Drought severity has an impact on the speed of gas exchange recovery (Brodribb and Cochard 2009). Severe droughts have effects on wood growth, which last several years after drought and still do not recover after an increase of water (Anderegg et al. 2015). Also, the timing of drought has been shown to impact the recovery, as droughts during the growing season led to longer recovery periods for growth (Huang et al. 2018). Additionally, rather than recovering to an identical state as predrought, plants may acclimate to survive future droughts. For example, reductions in growth after drought may be a form of drought acclimation‐ where plants make structural changes in xylem to bolster resistance to future droughts (i.e. smaller vessel diameters; Gessler et al. 2020; Gil‐Pelegrín et al. 2004). If reductions in growth are related to acclimation, then plants will have increased ability to survive a subsequent drought although with the trade‐off of lower long‐term carbon storage (Gessler et al. 2020).

Many studies use experiments to observe the physiological responses to initial drought stress but often do not observe the long‐term or repeated drought stress impacts on plant function (Miao et al. 2009; Gessler et al. 2020). It is critical to understand how multiple droughts will impact plant function because repeat droughts are predicted to occur with increasing frequency (IPCC 2014; Sperry et al. 2019). We used a common garden experiment to observe how aspen saplings respond to not just one, but multiple repeated droughts as well as two droughts separated by a wet recovery year. We asked: (1) How do multi‐year droughts impact hydraulic function and drought responses compared to single‐year droughts? (2) How will a well‐watered recovery year in between drought events impact hydraulic function and drought responses? (3) Do damages accumulate in the xylem, increasing aspen hydraulic vulnerability to drought, or do saplings exhibit hydraulic acclimation and improved resistance to future droughts? We hypothesise that: (1) Trees that experience repeat droughts exhibit reduced hydraulic function and increased physiological sensitivity to drought; (2) Trees that receive a well‐watered recovery period between drought events will exhibit recovery of hydraulic function yet intermediate physiological responses compared to trees that receive either the control or drought only treatments (i.e., trees with a recovery period between droughts will show improved drought tolerance during a second drought than drought only trees); (3) For trees in the consecutive drought treatments, xylem damages will accumulate, resulting in reduced hydraulic conductivity and drought resistance. Understanding how these aspen saplings respond to repeat droughts will be important to understanding how decreased hydraulic function impacts overall hydraulic vulnerability and likelihood of acclimating to climate change effects in the future.

## 2 Materials and Methods

### 2.1 Common Garden Experiment

We set up a multi‐year drought experiment at the University of Utah Biological Growth Facility in Salt Lake City, Utah, USA. The experiment took place in a common garden study that had been established as described in Kerr et al. (2023). The aspen saplings planted in the common garden had been grown from root samples collected in 2019 from five different aspen populations spanning a climate gradient across Utah and Colorado (Dixie, San Juan, Uncompahgre, White River and Uinta National Forests; for details on root collection, propagation and native site characteristics see Kerr et al. 2023). In 2020, propagules from the 5 populations were randomly planted amongst 10 beds and 36 propagules were planted in each bed with 50 cm of spacing on each side of the propagule (Supporting Information Figure S1). We mulched garden beds in 2020 and weeded the beds each spring before the drought treatments. Garden beds were irrigated with sprinklers to field capacity prior to drought treatments. While there were 5 different populations present, previous research found that most physiological traits did not differ by population (Kerr et al. 2023). Therefore, in this study, populations were pooled and only the treatment differences were investigated. While some populations were better represented overall, we used similar proportions of saplings from each population in each treatment group.

### 2.2 Experiment Setup

**Figure 1:**
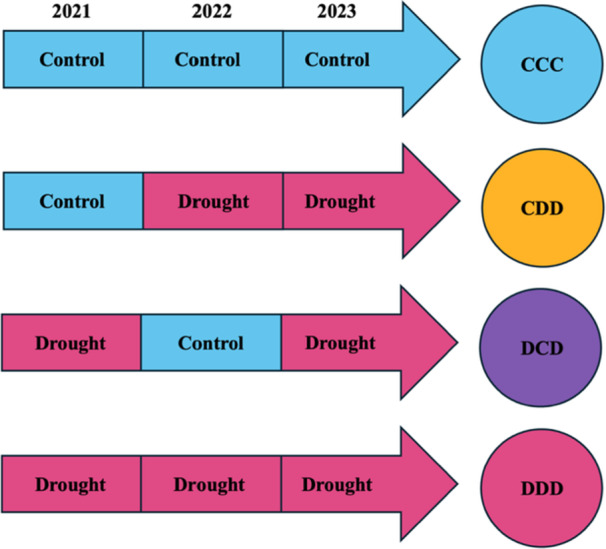
Overview of the four treatment groups. Arrows: The rectangle on the leftmost side of the arrow represents the watering treatment each treatment received in 2021, the middle rectangle of the arrow represents 2022 conditions, and the right most side of the arrow represents watering conditions in 2023. Well‐watered treatments are represented by blue boxes labelled “Control” while drought treatments are represented by pink boxes labelled “Drought.” Circles: The circles represent each of the four treatments where the first letter represents 2021 watering conditions, the middle letter represents 2022 watering conditions, and the last letter represents 2023 watering conditions, where C = control, and D = drought. The treatment colours are as follows: Blue = CCC, yellow = CDD, purple = DCD and pink = DDD.

The drought experiment ran for 3 years (2021‐2023). Each summer, different beds underwent a drought treatment resulting in four distinct treatment combinations after 3 years (Figure 1; Supporting Information Figure S1). In the first year (2021), 5 beds each were treated as control (C) and drought (D). In 2022, an additional two treatments were added. Two of the 5 control beds from 2021 remained controls (CC) while the other three were droughted (CD), and 2 of the 5 drought beds remained drought beds (DD) while the other three were well watered (DC; N of beds = 2 for CC and DD and N of beds = 3 for CD and DC). As the initial design of this common garden was not to maintain a multiyear drought (Kerr et al. 2023), the uneven split in the number of beds per treatment was unavoidable. We chose to include more beds in the alternating drought and water treatments to better constrain these largely‐understudied effects. In 2023, the DD, CD and DC beds were droughted (DDD, CDD and DCD respectively, with N of beds = 2, 3, 3, respectively) and the CC beds were well watered again (CCC, N of beds = 2). Drought treatments lasted for approximately 3 months each year (mid‐June to early September). The average VPD for each year during the drought treatment (June 1 – September 30) was 2.27 kPa in 2021, 2.22 kPa in 2022 and 1.86 kPa in 2023 (data calculated from air temperature and relative humidity using the plantecophys R package (Duursma 2015) from the nearby NHMU climate station (Synoptic Data 2026)). We aimed to reach predawn water potentials of −1.5 MPa for trees within the drought treatments as it is the average pressure at 50% loss in conductivity for aspen of these regions (Love et al. 2019) and reduced the amount of water applied through sprinklers or by hand watering accordingly. In 2021, drought‐treated beds received a 50% reduction in watering compared to the control treatment at first, further reducing that to 25% when the predawn leaf water potential target was not met (see Kerr et al. 2023 for more detail), resulting in a moderate drought effect. In 2022 and 2023, to ensure predawn leaf water potential targets were met earlier in the season and to obtain a more severe drought effect, drought treatments started out receiving 50% of the control's water and were gradually tapered down each week until they received only 2% by the final week. In 2022 and 2023, to prevent excess water from rainfall, we additionally placed pop‐up canopies above drought‐treated beds shortly before every major rainstorm (3 times in total), taking them back down within 12 h after each storm.

### 2.3 Drought Monitoring Measurements

Each year throughout the 3 months of drought treatment, we measured leaf water potentials and stomatal conductance (g_s_) weekly on a subset of 6 plants from each bed to prevent excessive defoliation of a single set of trees. Water potentials were measured using a pressure chamber (Model 1505D Pressure Chamber Instrument, PMS Instrument Company, Albany Oregon, USA) and stomatal conductance was measured using a steady state porometer (SC1 Leaf Porometer; METRE Group, Pullman, WA, USA). We measured leaf water potentials at predawn (4:00−6:00) and at midday (12:00−14:00), and stomatal conductance near midday (11:00−13:00). Additionally, at the end of the drought treatments in 2022 and 2023, we scored canopy mortality on all trees. We did this by visually estimating the proportion of the canopy that had died back by the openings left on branches by shed leaves or scorched and dead leaves left on the branches using methods described in Anderegg et al. (2019). We scored trees as dead if they had 100% loss in leaf area, exhibited signs of dead and splitting bark, and did not see new leaf sprout the following year. Trees that were dead in 2022 we did not include in the 2023 canopy mortality surveys.

### 2.4 Photosynthetic and Isotopic Measurements

Each year, we measured the photosynthetic response to intercellular CO_2_ concentrations (A/Ci curves) approximately 2 weeks after the drought treatment ended for the determination of the maximum rate of carboxylation (V_cmax_) and maximum rate of electron transport (J_max_). This 2‐week period between the cessation of the treatments and measurements was necessary as drought treated plants needed to recover stomatal conductance in order for us to measure photosynthesis. We measured A/Ci curves on an individual leaf of 10–13 trees per treatment using a LI‐6800 (Li‐Cor Biosciences, Lincoln Nebraska). To measure the curve, we initially set the CO_2_ concentration to ambient and decreased it stepwise to 50 ppm, brought it back to ambient, and increased stepwise until the curve reached a point where to photosynthesis appeared to no longer increase for the following CO2 ppm concentrations: 400, 300, 200, 100, 50, 5, 400, 400, 600, 800, 1000, 1200. Environmental conditions inside the LI‐6800 leaf chamber were set as follows: chamber temperature at 25°C, photosynthetic photon flux density at 2000 μmol m^−2^s^−1^ and relative humidity at ~55%. At each point in the A/Ci curve, the photosynthesis (A), g_s_, and concentration of CO_2_ in the intercellular space of leaves (Ci) were recorded. The plantecophys R package was used to fit the A/Ci curves to determine V_cmax_ and J_max_ (Duursma 2015).

Additionally, each year during the mid to late treatment period, we collected one leaf of every living tree (except 2021, when only 29‐35 trees per treatment were sampled) to measure the Carbon‐13 isotope, δ^13^C, which we used as a proxy for intrinsic water use efficiency (iWUE; i.e more negative δ^13^C are less efficient while less negative values are more efficient). Immediately upon sampling, we dried these leaves for at least 3 days at 60°C. We ground the dried leaves into a fine powder by flash‐freezing them with liquid nitrogen and grinding with a mortar and pestle. We then weighed and sealed ~ 0.7 mg of powdered leaf sample into small tin capsules. δ^13^C fraction was measured using an elemental analyser (Carlo‐Erba EA‐1110, TermoFinnigan, Bremen, Germany) with IRMS continuous flow interface (Finnigan Mat Delta + Conflo III, ThermoFinnigan, Bremen, Germany). The isotopic measurements were conducted by the Stable Isotope Ratio Facility for Environmental Research (SIRFER) lab at the University of Utah.

### 2.5 Hydraulic Measurements

Hydraulic conductivity measurements were made at the end of the drought treatments (peak drought) each year. We collected stem segments around 5–10 mm in diameter from 25 branches per treatment in 2021, 15 branches per treatment in 2022, and 6 branches per treatment in 2023. This reduction of hydraulic samples per treatment in 2023 was done because we did ring‐specific vulnerability curves, which are significantly more time consuming to perform (see methods below). We cut branch segments from the tree under water to prevent the introduction of embolism, wrapped the cut ends in wet paper towels, put samples in plastic bags, and placed them in a cooler for transport back to the lab. That same day, hydraulic sections were gradually cut down to 14 cm sections underwater with razor blades. The total leaf area downstream of the proximal cut was measured with a leaf area metre (LI‐3100C Portable Leaf Area Metre, LI‐COR Environmental Inc., Lincoln, NE, USA) and the leaf area to sapwood area (LA:SA) was calculated. We measured native hydraulic conductivity (K_native_) with a hydraulic apparatus (Sperry et al. 1988) using degassed, 0.2 um filtered, 10 mmol KCl solution. Following native measurements, we submerged samples in KCl solution and placed them in a vacuum chamber overnight to remove any preexisting emboli. We measured hydraulic conductivity again after vacuum infiltration to obtain the maximum hydraulic conductivity (K_max_).

Vulnerability curves were then measured to determine the vulnerability of the xylem to embolism (Tobin et al. 2013; Venturas et al. 2016). To construct vulnerability curves, we spun stems in a centrifuge at the following negative pressures: −0.5, −1, −2, −3, −4, −6, −8, −10, or until they reached at least 90% loss in conductivity from K_max_. We then calculated the PLC for each stem using equation 1 but correcting for xylem fatigue by making the conductivity at the −0.5 MPa spin the K_max_ (Hacke et al. 2001). We fit each curve to a two‐parameter Weibull function and extracted the 50% loss in conductivity (P_50_; Hacke et al. 2015).

(1) $$\text{PLC}(\%)=((K_{\max}*K_{h})/K_{\max})\;*\;100.$$

While we did not measure the maximum vessel length of our samples, it has been reported that 95% of the vessels in aspen are shorter than 5 cm and 1% are between 10 and 15 cm long, thus justifying the use of 14 cm stems for the centrifuge method (Sperry et al. 1994; Love et al. 2019).

At the end of the 2023 drought season, we measured ring‐specific hydraulic conductivity vulnerability using the protocol described in Fickle et al. (2025). In short, we used superglue to block off specific rings and measured the conductivity, then added more glue and measured the conductivity again, repeating the process until the conductivity was measured for the following bins of rings: the entire cross‐sectional area, 3‐year‐old ring, 2‐year‐old ring, and the newest ring. After hydraulic measurements were completed for each bin of rings, the superglue was cut off and the stem was spun in the centrifuge. We repeated this process for each spin, calculated fatigue‐corrected PLC and constructed vulnerability curves as described above, but for each bin of rings separately.

From the same stems used for ring‐specific vulnerability curves, we made thin cross‐section 2 cm from the proximal end of the stem using Teflon‐coated razor blades (GEM single‐edge stainless steel Teflon‐coated blades, Electron Microscopy Sciences, Haffield, Pennsylvania, USA). We mounted these thin sections on glass slides with glycerol and took images of each section using a dissecting scope (AmScope outlet 7X‐45X trinocular stereo zoom microscope, Irvine, California, USA) with an attached digital camera (Microscope Digital Camera MU500, AmScope, Irvine, California, USA). The area of each bin of rings was measured using ImageJ software (Schneider et al. 2012). The conductivity of each bin of rings was divided by the area of the ring‐specific xylem to get the xylem‐ring specific conductivity (K_s_).

### 2.6 Dye Perfusions and Anatomical Measurements

To determine how much active xylem was present in each ring, we conducted active xylem dye perfusions. In parallel to the ring‐specific hydraulic branches, we collected an additional set of 6 branches per treatment in 2023 for dye perfusions using the same procedure as described above. In the lab, we cut down branches to 10 cm in length underwater and shaved the ends with razor blades. We used the active staining technique (Jacobsen et al. 2007), where mild tension (~ −2 kPa) is applied to the distal end of branch segments to pull dye supplied from the proximal end through the branch segment. We used a 0.5% w/v Safranin O dye at a pH of 2. Dye was allowed to pull through the stems for at least 10 min or until dye was seen coming through the distal cut end, after which we promptly removed stems from the active staining system and placed them in a freezer to prevent additional dye bleeding. We made thin sections at 5 cm from the proximal end of the stems using Teflon‐coated razor blades and mounted them on glass slides with glycerol. Immediately after making each thin section, we used a dissecting scope with an attached digital camera to take images of the sections. To calculate the percent area of active xylem for each bin of rings, we measured the area of the same bin of rings as described for the ring‐specific hydraulics and the area of xylem stained red in each of these bins using ImageJ software.

From the same thin sections used for correcting the conductivity by xylem ring‐specific area, we took photos of vessels from each bin of rings using a microscope (Nikon Eclipse E600W, Model RT KE, Diagnostic Instruments) at 200x magnification with an attached digital camera (7.2 Colour Mosaic Camera, SPOT Imaging, Sterling Heights, Michigan, USA). Using ImageJ, we measured the area of at least 100 vessels per bin of ring. We calculated diameter of these vessels from the area assuming circular vessels.

### 2.7 Tree Growth Measurements

To measure annual stem diameter growth, we removed roughly half of the trees from the common garden in June 2024. We allowed the uprooted trees to air dry horizontally outside for several weeks. We collected the stem material approximately 5 cm above the root‐to‐shoot junction using a fine‐toothed hand saw. For any trees that had conjoined shoots, in which the splitting occurred around the root‐to‐shoot junction, we cut a few cm higher than the split. We did this to avoid distorted rings from multiple piths. For trees that had multiple stems, we used the largest one for our measurements. We sanded the cross‐section closest to the root to shoot junction first with a belt sander with 80 and 120 grit sandpaper and then by hand incrementally up to 1000 grit. We scanned images using a digital scanner at 4800 dpi (Epson Perfection v700 Photo, Epson America Inc, Long Beach, CA, USA). We used ImageJ to measure the diameter of the stem at the 2020–2021, 2021–2022, 2022–2023 and 2023–2024 growth ring boundaries. The Relative Growth Rate (RGR) for each year was calculated for diameter growth using equation 2, where S_1_ and S_2_ are the diameters measured from the stem at the beginning and end of the growing season, t_1_ and t_2_ respectively.

(2) $$\text{RGR}\;=\left[\frac{\log_{e}\;\left(S_{2}\right)-{\;\log}_{e}\;(S_{1})}{{(t}_{2}-t_{1})}\right].$$

### 2.8 Statistical Analysis

For weekly drought monitoring measurements, we compared predawn leaf water potentials, midday leaf water potentials and stomatal conductance independently for each year, using linear mixed‐effects models with the week of measurement, treatment, and their interaction as fixed effects, and tree nested within bed as a random factor. The predawn leaf water potentials were multiplied by −1 and log transformed to meet normality assumptions. We compared the proportion of canopy mortality across treatments and years using a linear mixed effects model with treatment, year, and their interaction as fixed effects and individual tree nested in bed as a random effect. To meet the assumptions of the model, the proportion of canopy mortality values were square root transformed.

We used linear mixed effects models (Bates et al. 2015) with treatment as a fixed effect and bed as a random effect to compare for differences in the V_cmax_, J_max_, δ^13^C, and yearly P_50,_ K_native and_ K_max_ calculated on the entire cross‐sectional area data. We tested for differences in V_cmax_, J_max_, δ^13^C, RGR, and entire cross‐sectional area P_50_, K_native_ and K_max_ across treatments for each year independently. For hydraulic measurements done on a yearly basis on the entire cross‐sectional area, we removed any P_50_ value outliers less than −4 MPa, as this is a biologically realistic threshold for aspen in this region (Love et al. 2018). P_50_ data were transformed as the absolute value and then logged to meet the assumptions of the model. We also compared the K_max_ for each treatment across years to determine if the xylem was being damaged, we used a linear mixed effects model with treatment, year, and the interaction between them as fixed effects and plant nested within the bed as random factors. For comparisons in the leaf area to sapwood area ratio across years, we used a linear mixed effects model with treatment and year, and their interaction as fixed effects and bed as a random effect.

For ring‐specific P_50_, we used a linear model (using lm function in R) with ring, treatment, and their interaction as fixed factors. For ring‐specific hydraulics, we extracted the P_50_ values from the vulnerability curves and removed outliers using the same −4 MPa threshold. To test for differences in the proportion of active xylem in each ring across treatments we used a linear mixed‐effects model with treatment, ring and their interaction as fixed effect with tree as a random effect. To meet the assumptions of the model, we added a value of 1 to each proportion and log‐transformed the data. To test for differences in vessel diameters across treatments and years, we used a linear model with treatment and year as fixed effects.

To test for differences in relative growth rate, we compared across treatments for each year independently. We used a linear mixed effects model with treatment as a fixed effect and plant number as a random effect. To meet the assumptions of the model, data from 2021 to 2023 was log transformed while data from 2022 was square root transformed.

For weekly drought monitoring measurements, relative growth rate and ring specific P_50_, we used Type III ANOVAS (using car package; Fox and Weisberg 2019) to make overall comparisons. For overall comparisons of the other measurements (entire cross‐sectional area K_native_ and K_max_, P_50_, active xylem area, Vessel diameter, V_cmax_, J_max_ and δ^13^C) we used Type II ANOVAS. For all ANOVA tests, if interaction effects were not significant, they were removed from the model and rerun (Zuur et al. 2009). For pairwise posthoc analyses, a sidak P‐value correction was applied to the following analyses: Ring specific P_50_, Leaf area: Sapwood area, active xylem area, vessel diameter, RGR and weekly drought monitoring measurements. All analyses were conducted using the R statistical environment version 4.4.2 (R core team 2024). We used emmeans (Lenth 2024) to perform all post hoc tests.

## 3 Results

### 3.1 Drought Monitoring Measurements

**Figure 2:**
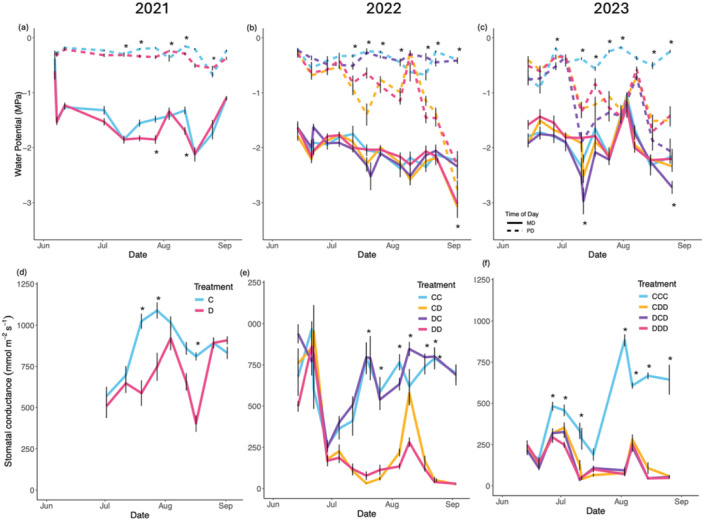
(a–c) Predawn (dashed lines) and midday (solid lines) water potentials measured during the drought treatments each year. (d–f) Stomatal conductance measurements during the drought treatments each year. Error bars represent the standard error. Both left panels are data from 2021, middle panels are data from 2022, and right panels are data from 2023. Stars signify weeks in which there were significant differences among the treatments. Specific post hoc pairwise analysis for significant weeks can be find in Supporting Information Table S2. [Color figure can be viewed at wileyonlinelibrary.com]

There were significant treatment effects that developed across the duration of the experiment in each year. For all 3 years, both the measurement week and the treatment‐by‐week interaction had significant overall effects on predawn leaf water potential. The treatment itself was only significant in 2023 (Figure 2a–c; Supporting Information Table S1). Differences between treatments were small in 2021, but more pronounced in the two subsequent years (Figure 2a–c, Supporting Information Table S2). In general, predawn leaf water potentials of droughted beds started dropping below the controls around treatment weeks 4–6 and remained lower for most of the remaining treatment duration, although not always significantly so (Supporting Information Table S2). Occasional peaks in predawn leaf water potential of the droughted beds were due to increased soil moisture from rain events (in 2022 and 2023, canopies were not always deployed in time). Notably, in 2023, aspen saplings in the treatment which received a well‐watered recovery treatment between the drought events (DCD, purple) experienced more negative predawn leaf water potentials during the subsequent drought and experienced differences earlier in the season (Figure 2c). Overall, in 2022 and 2023, the predawn leaf water potential target of − 1.5 MPa for drought treatments was reached, and more severe droughts were achieved.

Similar patterns were observed in midday leaf water potentials, though less pronounced. While the effect of treatment was not significant, both week and the treatment‐week interaction were significant, indicating that the treatment effect developed and increased over the growing season (Figure 2a–c, Supporting Information Table S1). Differences in midday leaf water potentials between treatments occurred mid‐drought (week 5) during 2021 and 2023, and during the last week in 2022 and 2023 (Figure 2a–c, Supporting Information Table S2). Similar to predawn water potentials, the treatment that received a well‐water recovery treatment between the drought events (DCD, purple) experienced lower midday leaf water potentials during the 2023 drought compared to the other treatments (Figure 2c).

While the overall effect of treatment was not significant, week and the interaction were indicating that drought stress elicited differences in stomatal conductance (Figure 2d–f, Supporting Information Table S3). The treatment effect itself was only significant in 2022. In 2021 and 2022, the stomatal conductance of the drought‐treated beds dropped below the controls starting at week 6 and generally remained lower for the duration of the experiment (Figure 2d–f; Supporting Information Table S4). In 2023, differences between drought and control treatments started around week 3 and generally remained so throughout the treatment. Peaks in stomatal conductance mid‐treatment were likely due to rain events as described above (early to mid‐August peaks).

For canopy mortality measured each year, there was a significant interaction between the treatment and year (F_3,677_ = 34.984, *p* < 0.001), and between treatments (F_3,677_ = 34.9941, *p* < 0.001), but not between years (F_1,677_ = 0.840, *p* = 0.360). Canopy dieback occurred most in years when treatments experienced drought conditions (Supporting Information Figure S2).

### 3.2 Photosynthetic and Isotopic Measurements

**Figure 3:**
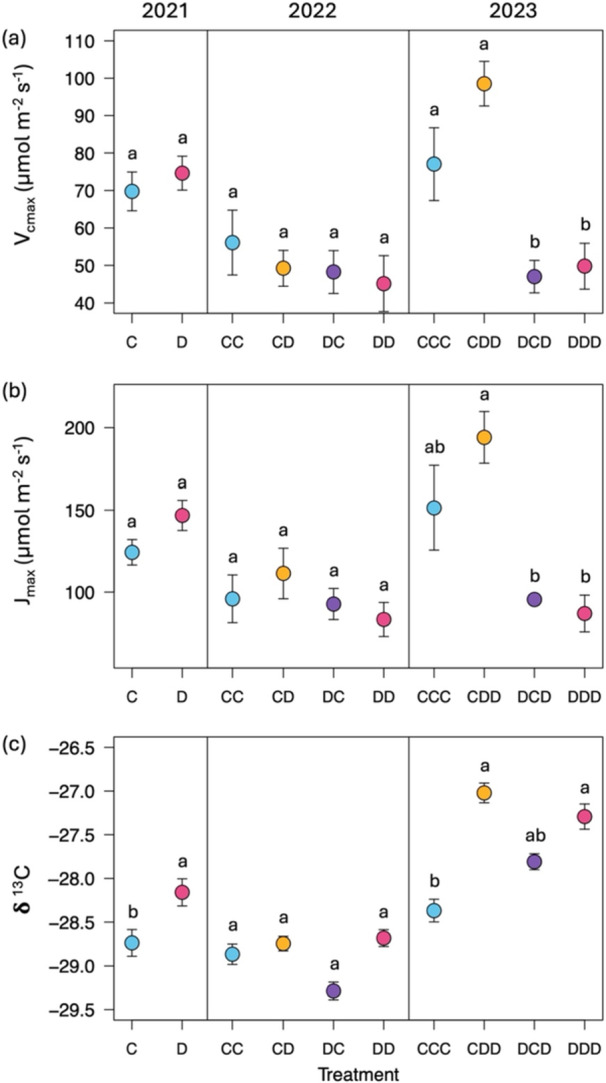
(a) V_cmax_ and (b) J_max_ measured two weeks after the drought period for each treatment each year. (c) The δ^13^C measured mid drought each year collected from leaf samples. For each panel, 2021 is on the leftmost side, 2022 is in the centre, and 2023 is on the rightmost side. Each point represents the mean, and the error bars represent the standard error. Lowercase letters represent significant differences within each year. [Color figure can be viewed at wileyonlinelibrary.com]

There were no treatment differences in V_cmax_ or J_max_ in 2021 (Figure 3a–b), however there were differences in δ^13^C, where the drought treatment indicates increased iWUE (Figure 3c, Supporting Information Table S5). There were again, no differences in V_cmax_ or J_max_ found in 2022 (Figure 3a–b). While there were no significant differences in δ^13^C between treatment groups in 2022, the treatment that received a well‐watered recovery year between drought events (CDC, purple) was lower, which indicates decreased iWUE compared to the CD and DD treatments (Figure 3c, Supporting Information Tables S5 and S6). In 2023, treatment had a significant effect on V_cmax_, J_max_ and δ^13^C. The control (CCC) and two consecutive drought treatments (CDD) had higher V_cmax_ than recovery (DCD) or three‐consecutive drought treatments (DDD), and the CDD treatment had higher J_max_ than the DCD and DDD treatments (Figure 3a–b). The control treatment has the lowest δ^13^C, indicating low iWUE, followed by the recovery treatment, with the two‐ and three‐ consecutive drought treatments indicating the most efficient iWUE (Figure 3c).

### 3.3 Hydraulics

**Figure 4:**
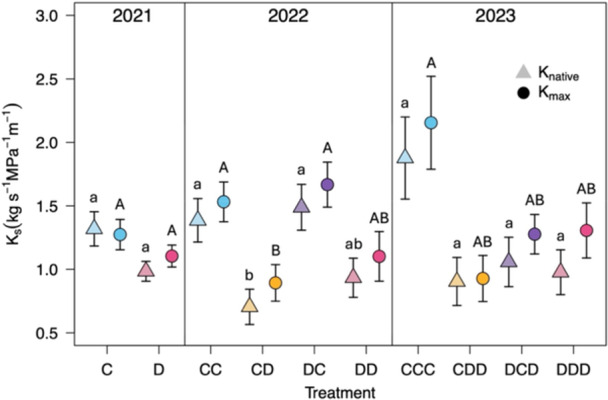
Stem area corrected hydraulic native (K_native_; Triangles) and max (K_max_; Circles) conductivity for each treatment within each year of measurement on the entire cross‐sectional area. Each point represents the mean and error bars represent the standard error. Lowercase letters represent significant differences within each year between K_native_ measurements and uppercase letters represent significant differences within each year between K_max_ measurements. [Color figure can be viewed at wileyonlinelibrary.com]

For hydraulic measurements on the entire branch cross‐sectional area, there was no effect of treatment on the P_50_ in any year (Supporting Information Table S7). In 2021 there were no differences between treatments on K_native_ or K_max_ (Figure 4, Supporting Information Table S8). In 2022 there was an overall effect of treatment on the K_native_ and K_max_ but pairwise comparisons only indicate a trend that the DCD treatment had both a higher K_native_ and K_max_ than the CDD treatment (Supporting Information Table S9). In 2023, treatment had an overall effect on both K_native_ and K_max_, but pairwise comparisons did not indicate any significance (Supporting Information Table S9). It should be noted that in 2023, there was a reduced hydraulic sample size. However, although the sample size declined, our statistical tests are robust to these effects and thus our results account for these changes in sample size.

**Figure 5:**
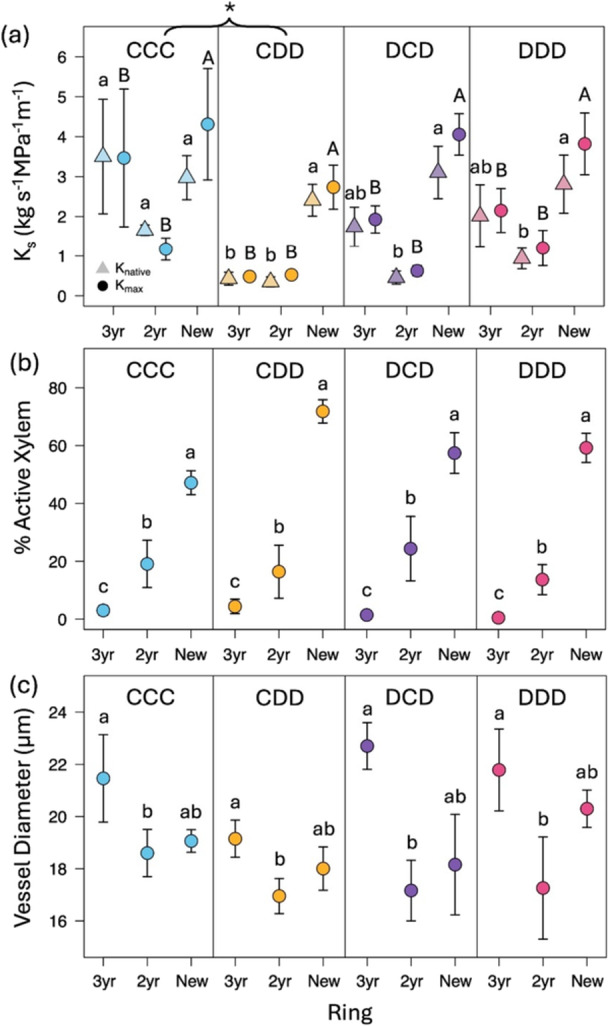
Ring specific measurements. (a) The K_native_ (triangles) and K_max_ (circles) for ring‐specific hydraulic conductivity, (b) the percent active xylem area for individual rings, and (c) the vessel diameters for each independent ring. Each point represents the mean, and the error bars represent the standard error. Along the X axis, the ring indicates the age of the independent ring measured, as all data in this figure was collected in 2023. 3 yr = ring from 2021, 2 yr = ring from 2022, and New is the current year ring in 2023. Letters signify significant differences. For panel (a), lowercase letters represent differences between the K_native_ while uppercase letters represent the K_max_. Additionally in all panels, letters only indicate differences across rings within treatments. Differences across treatments were not significant except in panel (a) where only CCC and CDD differ from each other (signified by a star on top of the panel. Note that for panel (a), the statistics were completed on log transformed data while untransformed data are presented in the graph. [Color figure can be viewed at wileyonlinelibrary.com]

For ring‐specific hydraulic measurements, both treatment and ring had a significant impact on K_max_, as the CCC treatment had a significantly higher K_max_ than CDD (Figure 5a, Supporting Information Table S10). Across all treatments, the newest ring always had the highest K_max_. For K_native_, there was not an overall impact of ring, however, there was an impact of the treatment, and an interaction between treatment and ring had a significant impact (Supporting Information Table S10). The CCC treatment had a higher K_native_ than the DCD treatment (Figure 5; Supporting Information Table S11). As for the P_50_, treatment (F_3,30_ = 2.734, *p* = 0.061) or ring (F_2,30_ = 0.840, *p* = 0.441) did not have a significant overall impact, however there were treatment effects in specific rings (Supporting Information Figure S3). Pairwise comparisons showed that, overall, all rings of the DCD treatment and the DDD treatment were more vulnerable than the CCC treatment as reflected in their P_50_ values (Supporting Information Figure S3; all *p*‐values < 0.05). Again, note the reduced sample size used for ring‐specific hydraulic measurements in 2023.

There was no impact of treatment on the leaf area to sapwood area ratio (LA:SA), however, the year and the treatment by year interaction had significant effects (Supporting Information Table S12). While there were no differences between the treatments in 2021 and 2023, in 2022, the control treatment had a higher leaf area to sapwood area than the CD treatment, and the recovery treatment (DC) had a higher leaf area to sapwood area ratio than both CD and DD (Table 13). Overall, the two consecutive treatments did not experience differences in their leaf area to sapwood area for any of the years, while the CCC and DCD did differ among years (Supporting Information Table S13). Both treatments showed a higher leaf area to sapwood area ratio in 2022 than in the other 2 years. Again, it should be noted in these comparisons that there was a reduced sample size taken in 2023.

### 3.4 Dye Perfusions and Anatomical Measurements

In the model comparing all treatments and rings, we found no overall significant effect of treatment on the percentage of active xylem in each ring. There were differences, however, in the percentage of active xylem in each ring, with the newest ring having the highest percentage of active xylem, followed by the 2‐year‐old ring, then by the 3‐year‐old ring (Figure 5b, Supporting Information Table S14). This pattern held true for all treatments. An independent analysis was done on just the newest rings, as there was a visible trend, and there was a significant difference between treatments (F_3,20_ = 3.7, *p* = 0.027), with CDD having a larger percent active xylem area than CCC (*p* = 0.015).

For all 3 years there was not a significant impact of treatment on the size of the vessel diameters of that year (Figure 5c; Supporting Information Table S15), however the vessel diameter did differ based on year, as across all treatments the vessels in 2022 were significantly smaller.

### 3.5 Tree Growth Measurements

**Figure 6:**
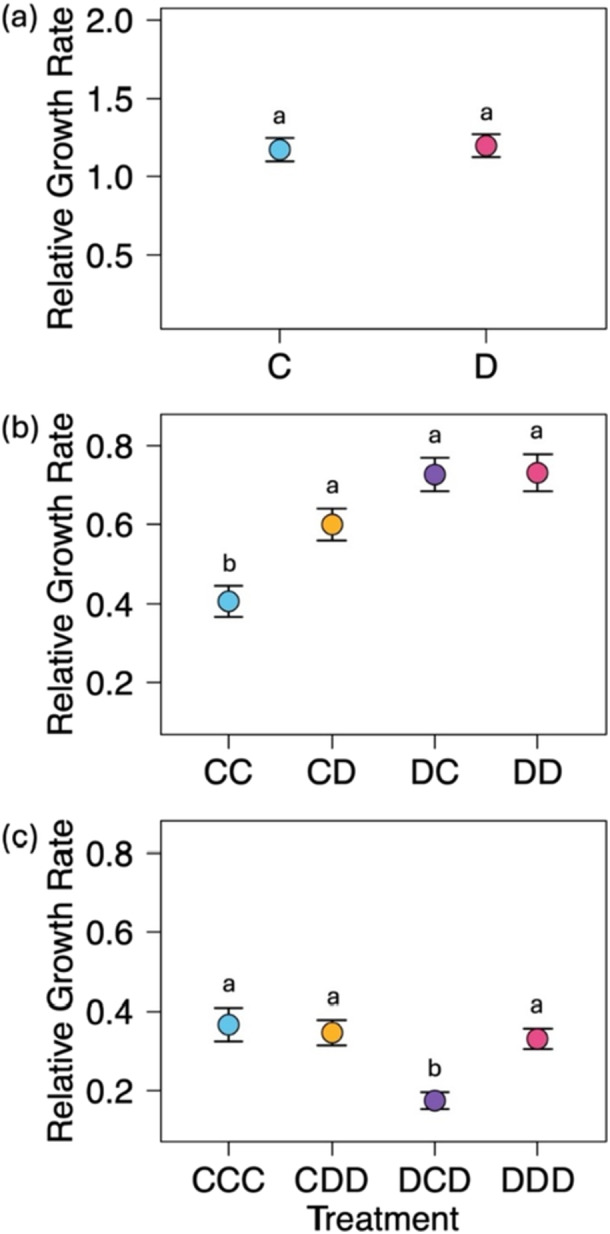
The relative growth rate for each of the treatments for each year. Lowercase letters represent significance. (a) In 2021 trees were split into two treatments, Control (C; blue) or Drought (D; pink). (b) In 2022 trees were split into 4 treatments Control‐Control (CC; blue), Control‐Drought (CD; yellow), Drought‐Control (DC; purple), or Drought‐Drought (DD; pink). (c) In 2023 there remained 4 treatments: Control‐Control‐Control (CCC; blue), Control‐Drought‐Drought (CDD; yellow), Drought‐Control‐Drought (DCD; purple), or Drought‐Drought‐Drought (DDD; pink). Note that the axis scale changes from plot a to plot b and c for ease of interpreting within year variation. Points represent the mean and error bars represent the standard error. [Color figure can be viewed at wileyonlinelibrary.com]

In 2021, treatment generally did not have an impact on the RGR, except that the CCC had significantly lower RGR than the other treatments. For both 2022 and 2023, treatment did have an impact on the RGR (Figure 6; Supporting Information Table S16). In 2023, the recovery treatment (DCD) experienced a significantly lower RGR than all the other treatments.

## 4 Discussion

Climate change is increasing the frequency of droughts substantially, yet the vast majority of forest ecophysiology studies and experiments tend to focus on single drought events. Thus, the potential for drought damage accumulation versus acclimation in trees over multiple droughts is a critical gap in the field. In this study we found little evidence of trees acclimating to drought stress over multiple years of drought. Contrary to our expectations, a recovery year between droughts tended to increase stress during a subsequent drought. Regarding our first hypothesis that trees experiencing repeat droughts would exhibit reduced hydraulic function and increased physiological sensitivity, all trees showed short‐term responses to drought in terms of the predawn water potentials and stomatal conductance. Additionally, we observed that droughted plants had reduced hydraulic conductivity but not hydraulic vulnerability as P_50_ did not change. Our second hypothesis that trees which receive a well‐watered recovery period would exhibit hydraulic function recovery was only partially supported, as during the year that the DCD treatment it experienced a recovery year, it experienced short‐term recovery in water potential and stomatal conductance but exhibited more extreme drought responses than the other treatments during the following years' drought, making it more vulnerable to drought. Finally, our third hypothesis that xylem damages will accumulate resulting in reduced hydraulic conductivity and drought resistance was only partially supported, as there was limited evidence that damages accumulated over multi‐year droughts. These accumulation of damages were apparent in the ring‐specific vulnerability curves as the DCD and DDD treatments had more vulnerable rings than the CCC treatment. These findings have important implications for understanding drought legacies and highlight the need for multi‐year‐repeated drought studies.

### 4.1 Multiple Droughts Impact Hydraulic and Physiological Function

Overall, in drought‐treated plants we observed extensive stomatal closure, likely to prevent excess water loss and to protect the hydraulic system from emboli (Hochberg et al. 2017). For aspen trees undergoing drought treatments, reductions in stomatal conductance occurred within the first few weeks of the drought treatments and stayed consistently lower throughout the remaining duration of the treatment. For these trees, the consistently low stomatal conductance resulted in higher intrinsic water use efficiency, as reflected in the less negative foliar δ^13^C values. Despite stomatal closure, photosynthetic traits remained broadly unaffected by the drought in 2022. However, in 2023, we did observe decreased J_max_ and V_cmax_ in the recovery treatment (DCD) and the tree consecutive years of drought treatment (DDD). Surprisingly, the treatment that experienced two consecutive droughts (CDD) had higher V_cmax_ and J_max_. We also observed significant leaf shedding and canopy dieback among the aspen trees in the drought treatments. Leaf shedding is commonly observed during severe droughts, as it allows trees to reduce their transpiring area and thus minimise their water loss (Wolfe et al. 2016; Li et al. 2019). In our case, however, most of the shed leaves showed scorch marks, indicating that the leaves may have overheated due to a lack of transpirational cooling (David et al. 2007; Pollastrini et al. 2019; Aparecido et al. 2020; Posch et al. 2024).

Despite the significant reductions in stomatal conductance and leaf area which serve to alleviate drought stress, we still observed reductions in the hydraulic conductivity of drought‐treated plants in 2022 and 2023. Specifically, the two treatments that consisted of consecutive droughts (CDD and DDD) had lower conductivity overall (Figure 4). However, we did not see differences in hydraulic conductivity between trees that experienced two consecutive drought treatments versus those that experienced three consecutive drought years. This might be partially explained by the relatively milder drought that the DDD treatment experienced in its first year of treatment. Both the CDD and DDD treatments experienced the same severe drought treatments in 2022 and 2023.

### 4.2 Recovery Year's Impact on Hydraulic Function and Drought Responses

For trees that experienced a well‐watered year in between two droughts, the predawn leaf water potentials, stomatal conductance, hydraulic conductivity and carbon isotopic discrimination were all similar to the control treatments in 2022, showing a clear recovery from the previous year's drought. However, during the second drought event the following year, the prior year's watering did not help those trees resist drought. Instead, in 2023, plants in the DCD treatment experienced more negative predawn and midday leaf water potentials, and lower relative growth rates compared to plants in the three other treatments. Additionally, in the ring‐specific vulnerability curves, trees in the DCD treatment experienced less negative P_50_ values in their two‐ and three‐ year‐old rings (Supporting Information Figure S3). While these two rings had overall lower conductivity than the newest ring, they are similar to those of the same age class in the other treatments (Figure 5a). The only measurement in which the prior year's watering appeared to make a positive impact is that the carbon isotope discrimination as the DCD was intermediate between the CCC and the consecutive drought treatments.

We hypothesise that this reduction in the health and more negative drought responses of the DCD treatment could be due to below‐ground responses to each year's treatment. During 2022 when the DCD treatment received a well‐watered recovery year, it is possible that the available soil moisture allowed the trees to recover their stomatal conductance, which allowed leaves to cool and possibly allowed the growth of additional non‐drought resistant roots and associates (mycorrhizae), in order to take advantage of the additional water. This idea is supported by the lower water use efficiency measured in this treatment in 2022 indicating possible growth. Additionally, the DCD treatment did not exhibit increased above‐ground growth compared to other treatments, indicating the possible prioritisation of below‐ground growth. Prior research has shown that trees metabolically prioritise their belowground structures during drought recovery years (Hagedorn et al. 2016; Gessler et al. 2020) as an increase in soil respiration follows drought release. In this study, however, when the following year's drought occurred, we hypothesise that it might have killed off all the new and vulnerable fine root growth and disconnected the trees from the soil‐ hence the reduced predawn leaf water potentials, reduced growth and increased hydraulic vulnerability (Cuneo et al. 2016). Additionally, aspen roots are particularly vulnerable to hydraulic failure, and root mortality is associated with drought‐induced stress (Anderegg et al. 2012b).

### 4.3 No Evidence of Hydraulic Drought Acclimation, Some Evidence of Damage Accumulation

We observed no evidence of drought acclimation in the xylem, as neither the entire cross‐sectional xylem P_50_ nor vessel diameters differed between the treatments for each year. Typically, xylem with smaller vessels is more drought resistant, and some studies have found trees to acclimate to drought by building smaller vessels (Gil‐Pelegrín et al. 2004). However, prior work has shown that vessel diameter is more responsive to water shortage early in the growing season rather than later, thus the timing of our drought treatment may have been too late to cause an effect (Hacke et al. 2017; Arend and Fromm 2007).

There was limited support for some accumulation of xylem damages, however this was mostly in the recovery treatment (DCD). The P_50_ for the ring‐specific measurements in the recovery treatment was significantly more vulnerable in the 2‐ and 3‐year‐old rings, indicating an acceleration of damage in the older rings. This was a surprising result, as we would have expected the repeat drought plants, rather than the ones given a recovery between droughts, to show signs of earlier xylem aging and damage accumulation. While not significant, there appears to be a trend of the K_max_ being lower in the second and third year in the CDD, and in the third year of the recovery treatment (Figure 5a). This implies that there are some sort of permanent blockages occurring, preventing vessel refilling despite flushing overnight. Permanent blockages such as gels and tyloses are associated with prior embolism events (Sperry et al. 1991; Taylor et al. 2002).

Instead of strong evidence of damage accumulation or acclimation, it appeared that trees undergoing drought closed their stomata and dropped their leaves to prevent hydraulic failure (Hochberg et al. 2017), and this was sufficient to prevent excessive damage to the xylem and accordingly negated the need for a strong acclimation response. While it would be expected that this strategy would have an impact on total stem growth, we did not see significant changes in their growth rates. Our drought treatments were applied in late June, with watering amounts reduced gradually and significant differences in predawn leaf water potentials often only occurring around July. This may have given the trees enough time to complete their growth early in the season before the peak‐season drought, which is not a surprising strategy for aspen, as many northern populations are heavily dependent on snowpack melt early in the season and less so on summer rains (Love et al. 2019). Alternately, bole growth may have been prioritised over other carbon pools even during drought stress, except in the DCD treatment where less above ground growth was observed, maybe due to below ground growth being prioritised. Additionally, we did see that the C treatment in 2021 had lower RGR. This, we consider, might be due to light competition within the control treatment beds as they were not limited in accessible water.

Several studies that have monitored multi‐year droughts did not agree on physiological acclimation. This is likely because of species‐specific traits and drought characteristics. For example, in a 2‐year drought study on European beech, it was found that trees that were irrigated after drought did not recover hydraulically (Tomasella et al. 2019). However, they did find that xylem traits differed based on the previous year's treatment, indicating some type of acclimation. Another study based on 3 years of consecutive drought found that one species of conifer experienced more xylem acclimation sensitivity than another conifer species, possibly due to one species ability to downregulate stomatal conductance (Oberleitner et al. 2022).

## 5 Conclusions

This study helps to address how young trees, which are the future of our forests, will respond to variable and repeat droughts. Filling these knowledge gaps about the physiological response of trees to repeat drought is crucial for our ability to predict how forests will respond to the changing climate and will help to increase modelling accuracy. In this study, we observed no evidence of hydraulic acclimation to repeated severe droughts. This will have far‐reaching implications for the response of aspen forests to projected drought frequency. Far from slowly acclimating to worsening drought regimes, our results suggest aspen forests may instead become increasingly vulnerable, as damages to the xylem accumulate over the course of repeat droughts. Additionally, we show that short recovery periods, such as a single wet year, may not directly benefit trees per se, instead potentially exacerbating their vulnerability even further. Future work should continue to track how hydraulic responses change under years to decades of drought and recovery and as trees age, so as to gain a more complete understanding of drought physiology.

## Supporting information

Supporting File

## Data Availability

The data that support the findings of this study are openly available in FIgshare at https://figshare.com/s/50bdb0e39b7c25a77d97.
