# Supplementary material for: Lack of Hydraulic Acclimation in Response to Multiple Droughts and Recovery
Source: Plant Cell Environ. 2026 Jun 15;49(9):6931–43. doi: 10.1111/pce.70671 (PMC13436473; doi:10.1111/pce.70671)
Supplement: Supplementary file 1 [file PCE-49-6931-s001.docx]

**Table S1.** Type III Anova results from the yearly predawn and midday leaf water potentials . TOD refers to the time of day: predawn or midday. Df refers to the degrees of freedom and significant P-values are bolded. N of individual measurements for each treatment in 2021 = 27-33, in 2022 = 9-19, and in 2023 = 8-18.

| TOD | Year | Comparison | χ^2^ | Df | P |
| --- | --- | --- | --- | --- | --- |
| Predawn | 2021 | Treatment | 0.850 | 1 | 0.357 |
|  |  | Week | 256.810 | 11 | **<0.001** |
|  |  | Treatment*Week | 85.271 | 11 | **<0.001** |
|  | 2022 | Treatment | 0.518 | 3 | 0.915 |
|  |  | Week | 68.508 | 11 | **<0.001** |
|  |  | Treatment*Week | 328.111 | 33 | **<0.001** |
|  | 2023 | Treatment | 13.206 | 3 | **0.004** |
|  |  | Week | 206.425 | 10 | **<0.001** |
|  |  | Treatment*Week | 478.549 | 30 | **<0.001** |
| Midday | 2021 | Treatment | 0.978 | 1 | 0.323 |
|  |  | Week | 176.422 | 11 | **<0.001** |
|  |  | Treatment*Week | 29.818 | 11 | **0.002** |
|  | 2022 | Treatment | 2.039 | 3 | 0.564 |
|  |  | Week | 50.479 | 11 | **<0.001** |
|  |  | Treatment*Week | 78.294 | 33 | **<0.001** |
|  | 2023 | Treatment | 5.445 | 3 | 0.142 |
|  |  | Week | 97.052 | 10 | **<0.001** |
|  |  | Treatment*Week | 71.359 | 30 | **<0.001** |

**Table S2.** Statistical results of the post hoc analysis: pairwise comparisons of leaf water potential measurements between treatments in weeks that had significant differences between at least two of the treatments in 2022 and 2023 based on the model in Figure 2 a,b,c. All other weeks contained only non-significant results and were therefore not added to the table. The first value for each pairwise comparison is the t-ratio, and the second value is the P-value. Significant P-values are bolded. TOD= Time of day (PD= Predawn water potentials, MD= Midday water potentials). Week refers to the week of measurement during the summer drought treatment, and the pairwise letters refer to the treatment.

| Year | TOD | Week | CC-CD | CC-DC | CC-DD | CD-DC | CD-DD | DC-DD |
| --- | --- | --- | --- | --- | --- | --- | --- | --- |
| 2022 | PD | 5 | -4.613, 0**.002** | -1.392, 1.000 | -3.128, 0.191 | 3.598, 0.0555 | 1.081, 1.000 | -2.052, 0.965 |
|  | PD | 6 | -8.194, <**.0001** | -0.696, 1.000 | -4.325, 0**.006** | 8.386, <**.0001** | 3.451, 0.082 | -4.043, 0**.014** |
|  | PD | 7 | -4.981, 0**.001** | -1.308, 1.000 | -4.922, 0**.001** | 4.054, 0**.013** | -0.413, 1.000 | -4.041, 0**.014** |
|  | PD | 8 | -3.860, 0**.025** | 0.044, 1.000 | -4.333, 0**.006** | 4.366, 0**.005** | -0.890, 1.000 | -4.792, 0**.001** |
|  | PD | 10 | -2.576, 0.616 | 3.292, 0.123 | -3.506, 0.070 | 6.480, <**.0001** | -1.264, 1.000 | -7.064, <**.0001** |
|  | PD | 11 | -7.755, <**.0001** | -1.512, 1.000 | -7.165, <**.0001** | 6.983, <**.0001** | -0.158, 1.000 | -6.343, <**.0001** |
|  | PD | 12 | -8.209, <.0001 | 0.183, 1.000 | -7.203, <.0001 | 9.154, <.0001 | 0.959, 1.000 | -8.046, <.0001 |
|  | MD | 12 | -4.272, 0**.002** | -0.692, 1.000 | -4.697, 0**.0003** | 3.983, 0**.006** | 0.308, 1.000 | -4.450, 0**.001** |
| 2023 | TOD | Week | CCC-CDD | CCC-DCD | CCC-DDD | CDD-DCD | CDD-DDD | DCD-DDD |
|  | PD | 3 | -2.541, 0.717 | -4.091, 0**.034** | -2.285, 0.891 | -1.717, 0.999 | 0.053, 1.000 | 1.603,  0.100 |
|  | PD | 5 | -5.207, **0.002** | -7.279, **<.0001** | -5.175, 0.002 | -2.202, 0.925 | -0.516, 1.000 | 1.420,  1.000 |
|  | PD | 6 | -2.775, 0.452 | -4.172, 0**.029** | -1.411,  1.000 | -1.015, 1.000 | 1.369, 1.000 | 2.570,  0.688 |
|  | PD | 7 | -6.974, **<.0001** | -7.760, **<.0001** | -7.137, **<.0001** | -0.863, 1.000 | -0.824, 1.000 | -0.048, 1.000 |
|  | PD | 8 | -9.210, **<.0001** | -9.470, **<.0001** | -8.811, **<.0001** | -0.291, 1.000 | -0.443, 1.000 | -0.182, 1.000 |
|  | PD | 10 | -5.005, **0.003** | -5.746, **0.001** | -5.172, **0.002** | -0.822, 1.000 | -0.583, 1.000 | 0.167,  1.000 |
|  | PD | 11 | -8.292, **<.0001** | -9.549, **<.0001** | -7.101, **<.0001** | -1.375, 1.000 | 0.406, 1.000 | 1.623,  1.000 |
|  | MD | 5 | 0.919, 1.000 | -3.561, **0.032** | 2.560,  0.527 | -4.952, **0.0001** | 1.881, 0.985 | 6.160, **<.0001** |
|  | MD | 11 | -1.183, 1.000 | -3.499, **0.038** | -0.281,  1.000 | -2.543, 0.540 | 0.826, 1.000 | 3.054, 0.155 |

**Table S3.** Type III ANOVA results from the yearly stomatal conductance measurements. Df = degrees of freedom and significant P-values are bolded. N of individual measurements for each treatment in 2021 = 27-33, in 2022 = 9-19, and in 2023 = 8-18.

| Measurement | Year | Comparison | χ^2^ | Df | P |
| --- | --- | --- | --- | --- | --- |
| Stomatal conductance | 2021 | Treatment | 1.100 | 1 | 0.294 |
|  |  | Week | 108.389 | 9 | **<0.001** |
|  |  | Treatment*Week | 62.418 | 9 | **<0.001** |
|  | 2022 | Treatment | 17.528 | 3 | **<0.001** |
|  |  | Week | 135.323 | 11 | **<0.001** |
|  |  | Treatment*Week | 422.137 | 33 | **<0.001** |
|  | 2023 | Treatment | 1.045 | 3 | 0.791 |
|  |  | Week | 778.783 | 9 | **<0.001** |
|  |  | Treatment*Week | 812.513 | 27 | **<0.001** |

**Table S4.** Statistical results of the post hoc analysis: pairwise comparisons of stomatal conductance between treatments in weeks that had significant differences between at least two of the treatments of 2022 and 2023 based on the model in Figure 2 d,e,f. All other weeks contained only non-significant results and were therefore not added to the table. The first value for each pairwise comparison is the t-ratio and the second value is the P-value. Significant P-values are bolded. Week refers to the week of measurement during the summer drought treatment, and the pairwise letters refer to the treatment (CC= Control-Control, CD= Control-Drought, DD= Drought-Drought, DC= Drought-Control).

| Year | Week | CC-CD | CC-DC | CC-DD | CD-DC | CD-DD | DC-DD |
| --- | --- | --- | --- | --- | --- | --- | --- |
| 2022 | 6 | 6.794, **0.0004** | 0.004, 1.000 | 5.925, **0.002** | -7.624, **0.0001** | -0.293, 1.000 | 6.507, **0.001** |
|  | 7 | 4.944, **0.010** | 0.184, 1.000 | 3.874, 0.096 | -5.284, **0.005** | -0.742, 1.000 | 4.043, 0.067 |
|  | 8 | 5.004, 0**.010** | 1.107, 1.000 | 5.125, 0**.007** | -4.359, 0**.037** | 0.664, 1.000 | 4.511, 0**.024** |
|  | 9 | 0.535, 1.000 | -1.769, 0.999 | 2.966, 0.480 | -2.582, 0.764 | 2.719, 0.668 | 5.047, 0**.009** |
|  | 10 | 5.424, 0**.004** | -0.398, 1.000 | 5.401, 0**.005** | -6.418, 0**.001** | 0.490, 1.000 | 6.245, 0**.001** |
|  | 11 | 6.776, 0**.0003** | -0.092, 1.000 | 6.197, 0**.001** | -7.668, 0**.0001** | 0.019, 1.000 | 6.872, 0**.0002** |
|  | 12 | 5.272, 0**.001** | 0.108, 1.000 | 5.529, 0**.003** | -5.595, 0**.0002** | -0.121, 1.000 | 5.941, 0**.001** |
| 2023 | Week | CCC-CDD | CCC-DCD | CCC-DDD | CDD-DCD | CDD-DDD | DCD-DDD |
|  | 3 | 3.921, **0.021** | 3.922, **0.021** | 4.134, **0.011** | 0.002, 1.000 | 0.572, 1.000 | 0.570, 1.000 |
|  | 4 | 2.511, 0.617 | 3.083, 0.190 | 4.563, **0.003** | 0.659, 1.000 | 2.432, 0.694 | 1.803, 0.993 |
|  | 5 | 5.186, **0.0004** | 6.318, **<.0001** | 5.667, **0.0001** | 1.184, 1.000 | 1.079, 1.000 | 0.046, 1.000 |
|  | 8 | 19.449, **<.0001** | 19.065, **<.0001** | 17.906, **<.0001** | -0.429, 1.000 | 0.165, 1.000 | 0.549, 1.000 |
|  | 9 | 7.838, **<.0001** | 8.850, **<.0001** | 7.806, **<.0001** | 1.193, 1.000 | 0.644, 1.000 | -0.444, 1.000 |
|  | 10 | 13.133, **<.0001** | 14.543, **<.0001** | 13.538, **<.0001** | 1.561, 1.000 | 1.432, 1.000 | 0.001, 1.000 |

**Table S5.** Type II Anova results from V_cmax_, J_max_, and δ^13^C between treatments for each year. Significant P-values are bolded. N of individual measurements V_cmax_ and J_max_ for each treatment in 2021 = 30-33, in 2022 = 4-7, and in 2023 = 4-6. N of individual measurements for δ^13^C for each treatment in 2021 = 30-34, in 2022 = 72-108, and in 2023 = 72-108.

| Trait | Year | Comparison | χ^2^ | Df | P-value |
| --- | --- | --- | --- | --- | --- |
| V_cmax_ | 2021 | Treatment | 0.433 | 1 | 0.511 |
|  | 2022 | Treatment | 0.621 | 3 | 0.892 |
|  | 2023 | Treatment | 29.877 | 3 | **<0.001** |
| J_max_ | 2021 | Treatment | 3.461 | 1 | 0.063 |
|  | 2022 | Treatment | 1.025 | 3 | 0.795 |
|  | 2023 | Treatment | 22.327 | 3 | **<0.001** |
| δ^13^C | 2021 | Treatment | 6.433 | 1 | **0.011** |
|  | 2022 | Treatment | 7.416 | 3 | 0.060 |
|  | 2023 | Treatment | 15.033 | 3 | **0.002** |

**Table S6.** Statistical results of the Post hoc analysis: pairwise comparisons of Vc_max_, J_max_, δ^13^C and between treatments for each year based on the model in Figure 3. The first value for each pairwise comparison is the t-value, and the second value is the p-value. Significant P-values are bolded.

| Year | Trait | CC-CD | CC-DC | CC-DD | CD-DC | CD-DD | DC-DD |
| --- | --- | --- | --- | --- | --- | --- | --- |
| 2022 | V_cmax_ | 0.414, 0.705 | 0.497, 0.644 | 0.083, 0.938 | 0.083, 0.938 | 0.375, 0.726 | 0.302, 0.774 |
|  | J_max_ | -0.757, 0.983 | -0.070, 1.000 | 0.196, 1.000 | 0.721, 0.986 | 0.911, 0.958 | 0.269, 0.999 |
|  | δ^13^C | -0.452, 0.667 | 1.621, 0.157 | -0.629, 0.553 | 2.307, 0.060 | -0.236, 0.821 | -2.299, 0.061 |
|  |  | CCC-CDD | CCC-DCD | CCC-DDD | CDD-DCD | CDD-DDD | DCD-DDD |
| 2023 | V_cmax_ | -2.073, 0.121 | 2.551, **0.039** | 2.714, 0.079 | 4.275, **0.003** | 4.704, **0.014** | -0.237, 0.820 |
|  | J_max_ | -1.692, 0.180 | 1.947, 0.093 | 2.635, 0.085 | 3.358, **0.010** | 4.246, **0.019** | 0.299, 0.774 |
|  | δ^13^C | -3.630, **0.011** | -1.542, 0.175 | -2.653, **0.039** | 2.328, 0.058 | 0.736, 0.489 | -1.357, 0.224 |

**Table S7.** Type III ANOVA results for the P_50_ data extracted from entire cross-sectional vulnerability curves measured each year. The output contains the Chi-squared test statistic (χ^2),^ the degrees of freedom (Df), and the P-value. Significant P-values are bolded. N of individual measurements for P_50_ for each treatment in 2021 = 26-30, in 2022 = 14-15, and in 2023 = 2-5.

| Year | χ^2^ | Df | P |
| --- | --- | --- | --- |
| 2021 | 1.510 | 1 | 0.219 |
| 2022 | 6.289 | 3 | 0.098 |
| 2023 | 3.137 | 3 | 0.371 |

**Table S8.** Type III Anova results for the K_native_ and K_max_ measured on entire cross-sectional area of branches each year. The output contains the Chi-squared test statistic (χ^2),^ the degrees of freedom (Df), and the P-value. Significant P-values are bolded. N of individual measurements for K_native_ and K_max_ for each treatment in 2021 = 29-33, in 2022 = 15, and in 2023 = 6.

| Year | Measurement | χ^2^ | Df | P |
| --- | --- | --- | --- | --- |
| 2021 | K_native_ | 3.663 | 1 | 0.056 |
|  | K_max_ | 1.253 | 1 | 0.263 |
| 2022 | K_native_ | 15.749 | 3 | **0.001** |
|  | K_max_ | 13.717 | 3 | **0.003** |
| 2023 | K_native_ | 10.305 | 3 | **0.016** |
|  | K_max_ | 13.701 | 3 | **0.003** |

**Table S9.** Statistical results of the Post-hoc analysis: pairwise comparisons between the treatments of the entire cross-section maximum conductivity (K_max_) and native conductivity (K_native_) for each year that there were significant treatment results based on the model in Figure 4. The first value is the t-value, and the second value is the P-value. There were four treatments: Control-Control-Control (CCC), Control-Drought-Drought (CDD), Drought-Control-Drought (DCD), or Drought-Drought-Drought (DDD).

| Trait | Year | CCC-CDD | CCC-DCD | CCC-DDD | CDD-DCD | CDD-DDD | DCD-DDD |
| --- | --- | --- | --- | --- | --- | --- | --- |
| K_native_ | 2022 | 2. 960, 0.165 | -0.447, 0.999 | 1.967, 0.555 | -3.410, 0.052 | -0.997, 0.933 | 2.416, 0.297 |
|  | 2023 | 2.744, 0.308 | 2.329, 0.374 | 2.542, 0.362 | -0.409, 0.999 | -0.207, 1.000 | 0.203, 1.000 |
| K_max_ | 2022 | 2.654, 0.232 | -0.564, 0.996 | 1.791, 0.633 | -3.221, 0.069 | -0.868, 0.9633 | 2.357, 0.317 |
|  | 2023 | 3.436,  0.203 | 2.410, 0.365 | 2.375, 0.440 | -0.989, 0.939 | -1.098, 0.918 | -0.085, 1.000 |

**Table S10.** Type III Anova results for the ring specific maximum conductivity (K_max_) and native conductivity (K_native_) measurements done in 2023. The output contains the Chi-squared test statistic (χ^2),^ the degrees of freedom (Df), and the P-value. Significant P-values are bolded. N of individual measurements for ring-specific hydraulic measurements for each treatment = 4-6.

| Year | Factor | χ^2^ | Df | P |
| --- | --- | --- | --- | --- |
| K_max_ | Treatment | 12.276 | 3 | **0.006** |
|  | Bin | 93.368 | 2 | **<0.001** |
| K_native_ | Treatment | 20.573 | 3 | **0.001** |
|  | Bin | 5.735 | 2 | 0.057 |
|  | Treatment*Bin | 17.727 | 6 | **0.007** |

**Table S11**. Statistical results of the Posthoc analyses: pairwise analyses between the different treatments for the pressure at 50% loss in conductivity (P_50_), the maximum conductivity (K_max_), and the native conductivity (K_native_) from the ring-specific vulnerability curves based on the models in Figure 5a and S3. The first value is the t-value, and the second value is the P-value.

| Trait | CCC-CDD | CCC-DCD | CCC-DDD | CDD-DCD | CDD-DDD | DCD-DDD |
| --- | --- | --- | --- | --- | --- | --- |
| P_50_ | 1.794, 0.394 | 3.258, **0.013** | 2.441, 0.108 | 1.716, 0.445 | 0.779, 0.969 | -0.939, 0.927 |
| K_max_ | 3.643, **0.010** | 1.222, 0.802 | 0.910, 0.940 | -2.558, 0.111 | -2.888, 0.056 | -0.330, 1.000 |
| K_native_ | 3.566, **0.012** | 2.082, 0.269 | 1.830, 0.405 | -1.565, 0.579 | -1.830, 0.406 | -0.265, 1.000 |

**Table S12.** Type III ANOVA results comparing the leaf area to sapwood area ratio across the different treatments, year of treatment, and the interaction between the two. The interaction effect is noted by a (*). The output contains the Chi-squared test statistic (χ^2),^ the degrees of freedom, and the P-value. Significant P-values are bolded. N of individual measurements for leaf area measurements for each treatment in 2021 = 29-33 , in 2022 = 15, and in 2023= 6-9.

| Factors | χ^2^ | Df | P |
| --- | --- | --- | --- |
| Treatment | 2.835 | 3 | 0.418 |
| Year | 23.466 | 2 | **<0.001** |
| Treatment * Year | 27.083 | 6 | **<0.001** |

**Table S13**. Statistical results of the Post-hoc analyses: pairwise comparisons between the treatments of the leaf area to sapwood area for each year. Each comparison contains the t-value followed by the P-value. Significant P-values are bolded. There were four treatments: Control-Control-Control (CCC), Control-Drought-Drought (CDD), Drought-Control-Drought (DCD), or Drought-Drought-Drought (DDD).

| Year | CCC-CDD | CCC-DCD | CCC-DDD | CDD-DCD | CDD-DDD | DCD-DDD |
| --- | --- | --- | --- | --- | --- | --- |
| 2021 | 0.195, 1.000 | -0.371, 1.000 | -1.352, 0.984 | 0.542, 1.000 | -1.459, 0.965 | -1.032, 0.999 |
| 2022 | 4.224, **0.005** | -1.719, 1.000 | 2.681, 0.244 | 5.047, **<0.001** | -1.559, 0.923 | 3.469, **0.037** |
| 2023 | -0.542, 1.000 | -0.185, 1.000 | 0.967, 0.999 | -0.341, 1.000 | 1.611, 0.882 | 1.157, 0.995 |

**Table S14.** Type III ANOVA results comparing the proportion of active xylem across different ring ages (Ring) and the treatments. The output contains the Chi-square test statistic (χ^2^) , degrees of freedom (Df), and the P value. Significant P-values are bolded. N of individual measurements for ring-specific dye measurements for each treatment = 4-6.

| Comparison | χ^2^ | Df | P |
| --- | --- | --- | --- |
| Treatment | 0.619 | 3 | 0.892 |
| Ring | 189.778 | 2 | **<0.001** |

**Table S15.**The ANOVA output comparing the vessel diameter across the different treatments and years. The output contains the test statistic F-value and degrees of freedom and the P-value. Significant P-values are bolded. N of individual measurements for ring-specific vessel diameter measurements for each treatment = 3-6.

| Comparison | F | P |
| --- | --- | --- |
| Treatment | F_3,58_ = 1.303 | 0.282 |
| Year | F_2,58_ = 10.283 | **<0.001** |

**Table S16.** Type III ANOVA results from the relative growth rate for each year independently. The output contains the Chi-squared test statistic (χ^2),^ the degrees of freedom (Df), and the P-value. Significant P-values are bolded. N of individual measurements for relative growth rate for each treatment in 2021 = 65-79, in 2022 = 26-45, and in 2023 = 25-44.

| Year | Comparison | χ^2^ | Df | P |
| --- | --- | --- | --- | --- |
| 2021 | Treatment | 0.024 | 1 | 0.877 |
| 2022 | Treatment | 27.665 | 3 | **<0.001** |
| 2023 | Treatment | 37.761 | 3 | **<0.001** |

Figure S1. Photographic documentation of the common garden experimental setup. a) Aerial image of the common garden beds, with each individual bed outlined with its respective treatment color. Treatments were as follows: CCC = Control-Control-Control, CDD = Control-Drought-Drought, DCD = Drought-Control-Drought, and DDD = Drought-Drought-Drought. Photograph sourced from Dale Forrister b) Photograph of a single garden bed containing 36 trees.

**Figure S2.** Percent canopy mortality (percentage of visual dieback of the entire crown) measured for each treatment in years 2022 (’22) and 2023 (’23). Points represent mean values, and error bars represent the standard error. Significant differences across all treatment groups and years are indicated by lowercase letters where significant results have unique letters. Across years, percent mortality was significantly different between treatment groups except for CDD and DDD. Treatments were as follows: CCC = Control-Control-Control in 2023 or Control-Control in 2022, CDD = Control-Drought-Drought in 2023 or Control-Drought in 2022, DCD = Drought-Control-Drought in 2023 or Drought-Control in 2022, and DDD = Drought-Drought-Drought in 2023 or Drought-Drought in 2022.

**Figure S3.** The pressure at 50% loss in conductivity (P_50_) for ring-specific vulnerability curves measured in 2023. The points represent mean values, and the error bars represent the standard error. Treatments are as follows: CCC = Control-Control-Control, CDD = Control-Drought-Drought, DCD = Drought-Control-Drought, and DDD = Drought-Drought-Drought. The panels separate the individual rings: 3yr = 3-yr- old ring, 2yr = 2-yr-old ring, New= current year ring. Rings did not differ within treatments but did across years. Note that the sample size for each treatment is 2-5 points. Significant differences between treatments within each ring are indicated by lowercase letters where significant results have unique letters.
